# Climatically Accelerated Material Processes Determining the Long-Term Reliability of Light-Emitting Diodes

**DOI:** 10.3390/ma17071643

**Published:** 2024-04-03

**Authors:** Gabor Harsanyi, Andras Poppe, Janos Hegedüs, Gusztav Hantos, Peter Bojta, Robert Kovacs

**Affiliations:** 1 Department of Electronics Technolgy, Budapest University of Technology and Economics, 3. Műegyetem rkp., 1111 Budapest, Hungary; 2 Department of Electron Devices, Budapest University of Technology and Economics, 3. Műegyetem rkp., 1111 Budapest, Hungary; poppe.andras@vik.bme.hu (A.P.); hegedus.janos@vik.bme.hu (J.H.); hantos.gusztav@vik.bme.hu (G.H.); 3 EFI-Labs Electronic Failure Analysis Ltd., 18. Egry J. u., 1111 Budapest, Hungary

**Keywords:** LEDs’ reliability and lifetime, ageing of LEDs, humidity accelerated processes in LEDs, spectral and luminous changes of LEDs

## Abstract

LEDs (Light-Emitting Diodes) are widely applied not only in decorative illumination but also in everyday lighting in buildings, flats, public areas, and automotive fields. These application areas often mean harsh environments, for example, regarding the humidity content of the surrounding air: besides outdoor and automotive illumination, even the household use cases (kitchen, bathroom, cellar) may represent extreme temperature and humidity variations (often reaching relative humidity levels close to 100%) for these devices; thus, their reliability behaviour in such circumstances should be better understood. Thermally activated processes were studied in several previous publications, but less information is available regarding high-humidity environmental tests. Moisture and temperature ageing tests with appropriate environmental parameter settings were performed as accelerated lifetime tests to investigate not only the effect of temperature but also that of humidity on the ageing and reliability of LED packages containing RGB (red green blue) chips and phosphor-converted white (pcW) LEDs. The ageing was followed not only through monitoring optical/electrical/spectral parameters but also with material analysis. Moisture–material interaction models were proposed and set up. It was found that humidity-accelerated ageing processes are more severe than expected from previous assumptions. RGB and pcW LEDs showed strongly different behaviour.

## 1. Introduction

LEDs (Light-Emitting Diodes) are widely applied not only in decorative illumination but also in everyday lighting in buildings, flats, public areas, automotive fields, etc. These application areas often involve harsh environmental conditions, such as the humidity content of the surrounding air. Besides outdoor and automotive applications, even households (kitchen, bathroom, cellar) may represent extreme humidity variations (often reaching relative humidity levels close to 100%) for LED packages. Thus, their reliability and behaviour under such operating conditions should be better understood.

The optical, electrical, and thermal parameters of LED packages continuously change and shift over time; these phenomena are a natural part of the device’s operation. During the first few hundred hours of operation, that is the so-called burn-in phase, in many cases, the emitted optical power radiant flux) and luminous flux can still be seen even to increase. Further ageing of the device is strictly associated with the deterioration of the optical parameters and with increasing heat dissipation. The two-volume book series co-edited and written by Willem Dirk van Driel and his co-authors [1,2] provides detailed information on the reliability of semiconductor-based light sources, starting from the component level and ending with a discussion of the possible failures of the system-level products. Pecht’s group published a Chapter within this book and a separate paper with a comprehensive study on the ageing of encapsulated LEDs [3]. Their study summarises the individual failure types of LEDs and their long-term and short-term effects. In a recent paper, a total number of 88 failure modes were reported, of which 60% are related to degradation. This indicates the importance of monitoring the degradation processes in these products, as longer lifetimes and warrantees are industry targets [4]. A large number of papers were published dealing with mainly the temperature ageing and voltage/current overload damages of the LEDs [1,2,3,4,5,6,7,8,9,10,11,12,13,14]. Much less research work was devoted to humidity-accelerated failure modes than to temperature-accelerated ones [15,16,17,18,19,20,21].

LED lifetimes are generally defined according to their ageing processes: LM-80 [22] is a standard that defines how the luminous flux output and colour shift of LEDs should be measured over time and at different temperatures [23]. It is supposed that catastrophic (suddenly appearing total) failure is not typical for LEDs, though many experiences show the opposite results: recycling containers are often full of failed (not operating) devices. The lifetime of LEDs is generally defined by the level of luminous flux loss (also known as luminous flux maintenance): 30% is often given as the critical maximum acceptable level; over this level of loss, the device is usually regarded as failed [24]. In a wider context, the lifetime of LEDs is defined statistically, for a given population of LED packages: the luminous flux maintenance is considered for the entire population: the ageing time at which the light output shrinks below *x* percent of the initial value for *y* percent of the LED packages in the entire population is called the L*x*B*y* lifetime. e.g., for everyday applications, the L70B50 lifetime is the number of operating hours at which the luminous flux shrinks to 70% of the initial value in the case of 50% of the entire LED population. Also, the colour coordinate and spectral changes could be critical. In the case of horticulture applications for example, instead of the luminous flux maintenance, a better, more relevant ageing inductor would be the maintenance of the total photon flux. Overall, regardless of the actual ageing indicator, the ageing depends strongly on the operating temperature that is determined by the ambient temperature, the properties of the heat exchange with the environment, and the power dissipation of the device [6,8,13,14]. The latter can be controlled with a smart supply and with appropriate experimental data the operating parameters can be optimized for getting lifetimes as long as possible; also, a prediction can be calculated for the “remaining useful lifetime” [14,16]. Thermally activated processes can be described very well by the Arrhenius-type lifetime model and temperature-dependent acceleration factors can be calculated: lifetime predictions can be estimated with accelerated lifetime tests [8,11,20]. This is, however, a solely temperature-based model, not calculating with other environmental conditions, such as chemical (humidity) parameters. The standard also requests performing the measurement tests under 65% relative humidity [21], which means a relatively “low” level and environmental ageing tests should be made at higher moisture levels. Even the measurement parameters can be influenced by the humidity, as described in [21].

The failure mechanisms of LEDs are divided into three categories based on the failure sites: semiconductor, interconnect, and package [1,2,3,4]. Semiconductor-related failure mechanisms include defect and dislocation generation and movement, die cracking, dopant diffusion [6,7,8], and electromigration [5]. Interconnect-related failure mechanisms are electrical overstress-induced bond wire fracture, wire ball bond fatigue, electrical contact metallurgical interdiffusion, and electrostatic discharge [9,10]. Package-related failure mechanisms in LEDs include carbonization or yellowing of the encapsulant [3,7,18,19], delamination, lens cracking [7,17], phosphor degradation [7,16,19,21], and external solder joint fatigue, which is out of the scope of the device-level studies. Wu [12] (p. 1300) and co-workers found that in a “high-temperature environment, epoxy resin occurred serious carbonation and the extraction efficiency of blue LED modules decreased sharply. But the methyl silicone resin and phenyl silicone resin showed excellent thermal stability, especially the former one”. Nowadays, SMD (Surface Mounted Devices) and filament COB (Chip on Board) LEDs generally apply polysiloxanes for cover films, also embedding phosphors.

The majority of LEDs’ failure modes are thermally activated and fit well into the Arrhenius-type lifetime model [1,2,3,4,8,11,20] (discussed in the standard JEDEC JESD91A) [25]. The acceleration factor (*AF*), the mean time to failure (*MTTF*) ratios in the usage (index *u*) and test (index *t*) can be expressed as

(1) $$AF={MTTF}_{u}/{MTTF}_{t}=exp\left[\frac{E_{a}}{k}\left(\frac{1}{T_{u}}-\frac{1}{T_{t}}\right)\right],$$

where *T* is the temperature, *E_a_* denotes the activation energy of the process, and *k* is Boltzmann’s constant. These types of acceleration models generally only apply to one specific failure mechanism and do not apply to a system-level estimate of life. If one failure mechanism is the dominant one for the product, then that mechanism would provide a life estimate. Otherwise, parallel processes take place concurrently (as in the case of LEDs as listed above), and the fastest process will determine the lifetime; many times evolving randomly.

As mentioned before, less research work has been devoted to humidity-accelerated failure modes. Some of them were described based on the hygro-mechanical stress [3,17]. The absorption of moisture can induce hygroscopic swelling in LED packaging materials such as moulding polymer, die attachment, and epoxy lens. The hygroscopic swelling linearly increases with moisture content. The different levels of swelling induce hygroscopic stress in the package, thereby inducing delamination. As moisture continues to enter into the die attached material, delamination at the die-attach will occur and thus increase the thermal resistance of the dice and reduce the lumen output. Test results from the study of Lall and co-workers [16] also demonstrated that humidity can alter the phosphor and binder layer that is on top of the photon emitter. Other studies practically strengthened the results that humidity causes mostly the degradation of the phosphor and its sealant matrix [18,19,20,21], but the material interactions behind the degradation are explained contradictory: epoxy carbonization [12], refractive index shift of silicone [19], hydrolysis, and the related transmittance change of the polysiloxane [18]. All studies agree that humidity absorption results in the degradation of the light-conversation of the phosphor [16,17,18,19,20,21]. Thermal Humidity Bias (THB), otherwise called MET (Moisture, Electric, Temperature) stress tests were proposed as accelerated lifetime tests to investigate not only the effect of temperature [3,4,20,21]. The test conditions were proposed mainly according to the JEDEC standards [26]: 85 °C/85%RH (Relative Humidity) that is often applied for testing electronic devices, assemblies and appliances. The failure modes are often similar to the thermally activated ones, but there are different mechanisms behind them. It must be emphasized, though, that humidity-induced processes are also temperature-dependent—as practically all phenomena in nature. Huang and co-workers found that a luminous flux loss of almost 10% can be experienced with white LEDs after 1000 h ageing at 95 °C/95%RH [20], which seems to be worse than expected. A similar conclusion was made by Shingh and Tan [21] (p. 11): “White LEDs (i.e., with the presence of phosphor) show rapid percentage lumen degradation as compared to blue LEDs (without phosphor) when tested under MET test. RGB LEDs without phosphor integrating 3 colours red, green and blue to produce white light could be a better option than white LEDs having blue or UV LEDs with phosphor. However, no reliability study on such RGB white LEDs had been reported until that time thus, the necessity of the MET test was also demonstrated”. They also stated that “the presence of phosphor in white LEDs provides protection for LED chip from moisture as the heat accumulation in white LEDs phosphor evaporates the moisture from the LED die surface” [21] (p. 12).

The acceleration factor (AF) estimation at humidity-induced ageing processes needs the extension of the Arrhenius model. Peck and colleagues gathered reports and studies of the effects of temperature and humidity on the time-to-failure behaviour for a range of epoxy-based packages. The model they developed is empirically derived from a large number of different life studies [27]:

(2) $$AF={\left(\frac{{RH}_{u}}{{RH}_{t}}\right)}^{-n}exp\left[\frac{E_{a}}{k}\left(\frac{1}{T_{u}}-\frac{1}{T_{t}}\right)\right],$$

where *RH* is the relative humidity and *n* is an empirical constant. The constants were reported as *n* = 2, 7… 3, E_a_ = 0.8–0.9 eV [28]. It must be emphasized that this relationship is an acceleration model for temperature and humidity evaluation of moisture-induced IC failure within epoxy over-moulded components. This is a structure very similar to that of the LED packages. However, it is not a general model for any application of temperature and humidity testing. However, Huang and co-workers applied this model for LED ageing with success [20].

We have some criticism for the application of this model. Equation (2) shows that the temperature and humidity dependence can be separated in the function. If we consider the definition of relative humidity,

(3) $$RH=\frac{p_{w}}{p_{s}},$$

where *p_w_* is the actual and *p_s_* the saturated partial pressure of water vapour at the given temperature, which can be expressed from the Clausius–Clapeyron equation as

(4) $$p_{s}=p_{0}exp-\left(\frac{L}{RT}\right),$$

where *R* is the specific gas constant (i.e., the gas universal constant divided by the molar mass, *T* is the absolute temperature, and *L* is the specific latent heat (or specific enthalpy) of vaporization. The conclusion is that the relative humidity is not independent of the temperature, their ratio itself will also contain an Arrhenius-type exponential function of the temperature. If one would like to separate the effect of temperature and humidity, we suggest to reformulate Equation (2) as follows:

(5) $$AF={\left(\frac{H_{u}}{H_{t}}\right)}^{-n}exp\left[\frac{E_{t}}{k}\left(\frac{1}{T_{u}}-\frac{1}{T_{t}}\right)\right],$$

where *H* is the absolute humidity concentration and *E_t_* is an energy dimension parameter containing the sum of activation energies from several processes.

As mentioned earlier, in the case of LEDs a given level of the reduction in light output—not a catastrophic failure, as in other electronic components—is the usual failure criterion. Failure mechanisms to cause a catastrophic failure are considered for system-level life prediction. In practice, however, a large number of LED lighting systems operating in the field suffered from the catastrophic (suddenly appearing) failure type that was considered to be the failure of the supplying electronics. A large portion, however, belongs to the NFF (No Faults Found) category indicating that the LED devices themselves may also be responsible for such types of catastrophic failures. The general experience with electronic assemblies and components is that they can survive JEDEC JESD22-A101D.01 “Steady-State temperature-humidity Bias Life Test” (85 °C/85%RH) [26] with no faults, but in many cases, early mode catastrophic failures appear in the field. The reason is that the weather changes can produce extremely high humidity levels even at relatively low temperatures and the dew point conditions can be reached. Continuous moisture (sometimes only monomolecular) films can be formed on the device surfaces far below dew point humidity levels depending on the surface conditions and contaminants. The moisture can penetrate the package through cracks and pinholes and then reach chip-level depths, causing reduced insulation resistance and inducing corrosion/migration type processes resulting in massive short circuits or openings, or other processes in the case of LEDs that are unrevealed or not clearly described yet (delamination, phosphor degradation, see above).

**Figure 1:**
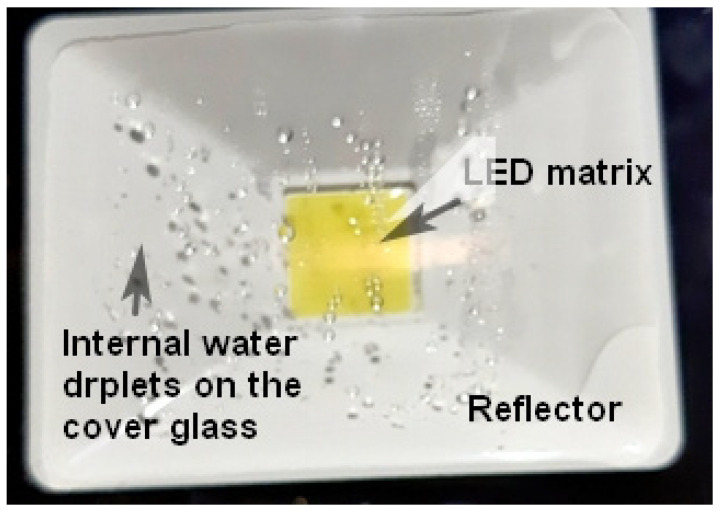
Hermetically closed inert gas-filled IP65 type outdoor reflector illustrates internally closed moisture precipitation at harsh environmental effects.

Nowadays, manufacturers advertise their products as waterproof while also giving the IP code. The IP code (Ingress Protection Code) is defined in the IEC 60529 standard [29], which classifies and rates the degree of protection provided by mechanical casings and electrical enclosures against intrusion, dust, accidental contact, and water. Waterproofness categories are defined against dripping, spraying, splashing and jet water and appropriate tests can be performed. Pure SMD LED devices belong to the IP20 category, which means (the second number 0) practically no qualification against water. Lightbulbs and indoor applications also belong to this group. Lightbulbs are closed mechanically or by glueing, which does not mean water-tight sealing and they do not contain special internal gases. Automotive light “bulbs” do not even apply real coverage; the SMD devices are in direct contact with the environment or the atmosphere of the lamp enclosure. Many types of lighting or decorative strips also contain unprotected SMD LEDs. Outdoor devices and strips apply some kind of water-resistant sealing or plastic cover on the strips and belong to the IP65–IP68 categories. The waterproofness, though, is typically checked in the initial stage of the products and may change during the ageing of the applied materials. Even waterproof constructions may change their properties and may become permeable to water molecules at high humidity levels after some period of work. Even hermetically closed inert gas-filled constructions may fail from moisture precipitation if the internal component surfaces or the inert gas filler itself contained some amount of humidity before sealing the device. An IP65-type outdoor reflector shown in Figure 1 illustrates these phenomena very well.

A highly accelerated temperature and humidity stress test (HAST: JEDEC JESD22A110E-01, [30]) and steady-state temperature-humidity Bias Life Test JEDEC JESD22-A101D.01 (THB, 85 °C/85%RH, called shortly 85/85 test [26]) and other environmental ageing tests can help to reveal the potential failure problems in these cases.

In our previous study, HAST tests were performed on 5050-type phosphor-converted cool white (pcCW) SMD LEDs [31]. The conditions were 105 °C, 100%RH, 1.2 atm, under ½ max. power conditions (ca. 10 mA pro chip, 3 chips in package), the duration was 300 h for checking electrical/thermal/optical parameters, and it was continued for a total duration of 1000 h in order to see all degradation processes.

The main experiences were as follows: thermal resistance to the environment changed slightly in the range of accuracy, which could be explained by the degradation (or delamination) of the chip attachment glue, as described elsewhere [17], and should be strengthened by further tests. The junction temperature increased also slightly. Dissipated (thermal) power increased significantly and a strong decrease in the optical emission power was detected at the same time. This may be the result of the degradation of the chip [3,5], that of the phosphor, or the darkening of the transparent and reflective layers [2,4,5,21]. The devices have to be regarded as “failed” (according to the LM-80 standard and related manufacturer requirements [2,4,5,21]) since the loss of the original luminous parameters is far over the acceptable level (30 or 50%). The shift of the calculated colour temperature towards the blue also indicated the degradation of the (yellow) phosphor. The corrosion analysis on the aged and decapsulated LEDs showed that the reflecting surface finish Ag of the leadframe is not present in the precipitate between the metallisation stripes, and thus no Ag migration occurred. However, Fe was definitely present and non-conducting Fe_2_O_3_ was probably formed; the Fe originated from the Alloy 42 lead frame substrate metal material.

For a further better understanding of the possible failure modes of LEDs and LED-based lighting instruments caused by high humidity levels, tests in appropriate conditions and subsequent failure analyses were performed on the selected LED types described subsequently.

## 2. Samples and Methods

For the experimental work, a good representative sample type had to be selected. A huge number of LED-based lighting systems (various light bulbs, light tubes, lamps for indoor and outdoor applications, LED reflectors, automotive “bulbs”, as well as light strips use white packaged SMD LEDs (like 5050) and similar package constructions (5630, 2835, 3014, 2216, 3530, 3030, etc.). Even filaments in “nostalgic” retrofit lightbulbs and Multichip Modules in reflectors have very similar geometrical and material structures. In these devices, the chips are mounted by chip attachment glue onto a substrate or lead frame, and wire bonding is used for contacting the chip pads. In SMD devices, the chips are placed into plastic cases equipped with the necessary metallization lead frame, and the small pool in the middle is filled with transparent PDMS or epoxy that contains the necessary phosphor. White LEDs operate generally with blue LED chips covered by a yellow phosphor; a commonly used phosphor type is YAG:Ce. RGB LED packages contain three different chips (red, green, and blue) covered with a transparent mould material without any phosphor.

**Figure 2:**
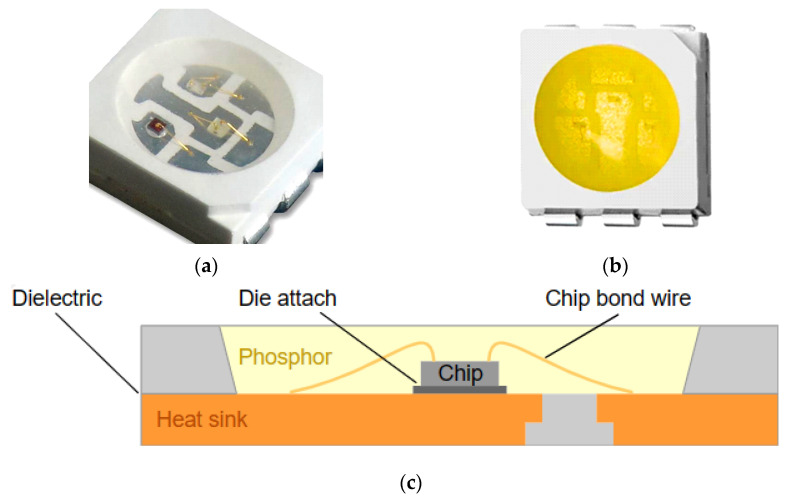
Images of the 5050-package-type SMD LEDs: (**a**) RGB LED, (**b**) phosphor-converted white LED, (**c**) cross-sectional view of the package structure.

Considering all the abovementioned aspects, a 5050 SMD LED package was chosen. Such 5050 package types are available as cool and warm white devices as well as RGB colour LEDs, in which case the package contains three LED chips. (These LED packages do not contain any lens for beam forming). Figure 2 shows images of such LED packages.

**Figure 3:**
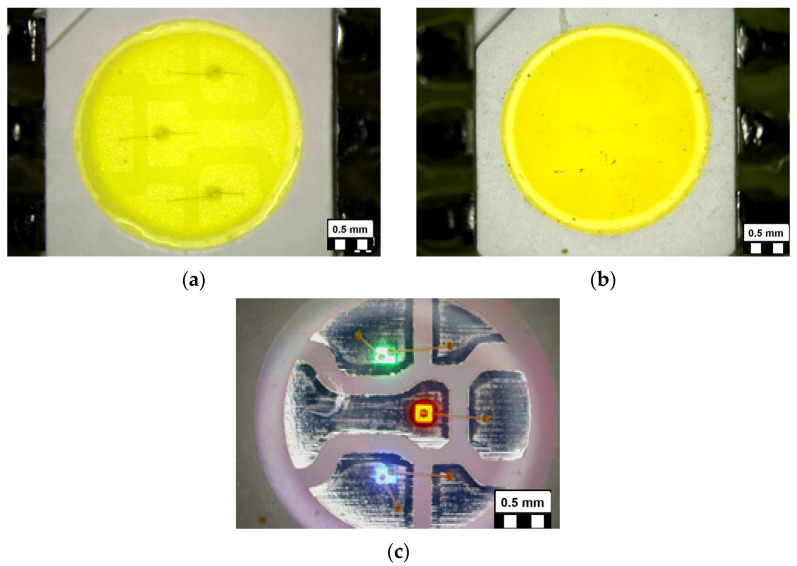
Microscopic images of the investigated 5050-package-type SMD LEDs: (**a**) pc cool white, (**b**) pc warm white, (**c**) RGB type (bar unit in the corners indicate 0.5 mm).

The microscopic photograph pictures of the used sample types are shown in Figure 3. On the cool white (CW) LED, the outlines of the chips with their under-laying attachment material droplet, their bonding wires, and the lead frame metallization system are recognizable through the yellow phosphor–polymer composite. The warm white (WW) samples are significantly less transparent since the concentration of the yellow phosphor particles is higher in the phosphor–polymer composite. The transparency of the RGB LED’s cover material is excellent. Blue and green chips use two bonding wires each, indicating insulating chip substrate material (generally, a multilayer InGaN/GaN semiconductor structure is deposited on a structured sapphire substrate), while in the case of red chips GaP/AlInGaP is deposited onto Si substrate [1,2,3,4,5,6,7,8,9,10], with the bottom of this substrate forming one electrical contact, necessitating only a single bonding wire on the top.

The THB 85/85 (Thermal Humidity Bias) Test (JEDEC JESD22-A101D.01, Steady-state Temperature Humidity Bias ageing test [26]) was planned with the following conditions:

1. Temperature: 85 °C, 85% RH, atmospheric pressure, under ½ max. power conditions according to datasheets (ca. 10 mA pro chips) (Weiss Chamber WB3 I 80/40, product of Weisstechnik, Balingen, Germany);
2. Duration: 150, 250, 500, 800, 1000 h ageing;
3. Samples: pcCW, pcWW, and RGB 5050 SMD LEDs, 50 pieces of each type containing three chips/package, as shown in Figure 3. (All parts of the interconnection system on which the LEDs are mounted and soldered for the tests: printed circuit interconnection boards, soldered connections, and also those of the LED packages and connecting wires were covered by a silicone protective layer in order to avoid migrated shorts or other corrosion failures outside of the LED packages);
4. Continuous visual inspection of all packages was performed during the ageing; samples were taken periodically for electrical/optical measurements;
5. The overall measurement and test setup was (as described in previous studies [13]) a combined electrical, thermal, and radiometric/photometric measurement system complying with the recommendations of the electrical test method as per JEDEC JESD51-1 [32], JEDEC JESD51-51A and JESD51-52A standards [33,34], and the total flux measurement method for LEDs according to the CIE 225:20172007 document [35]. In such a setup the electrical powering of the LED under test is provided by the thermal test equipment. The light output of the LED is measured when it is driven by its prescribed operating current and is in thermal equilibrium (steady-state) as required by [35]. The reference temperature of the LED under test is the temperature of the cold plate which is attached to the integrating sphere of the photometric test system and the LEDs’ junction temperature is determined as recommended in [33]. For a schematic diagram and a detailed description of such a test setup, refer to [36];
6. Optical microscopy (OM), scanning electron microscopy (SEM), and related electron dispersed X-ray (EDX) analysis and other microanalysis methods (see later) were used to reveal the background of the ageing processes resulting in the parameter changes of the LEDs and leading to possible failure.

## 3. Results

The main results of the 85/85 environmental ageing tests are summarized below. It must be emphasized, that under these circumstances, the 85 °C ambient temperature means a very harsh thermal environment, resulting in high junction temperatures during ageing, and thus, all processes are accelerated by the temperature as well. Relative humidity of 85% means a moderate level though, taking into consideration that humidity levels even over 95% may often occur in outdoor environments. Humidity and temperature-driven acceleration cannot be distinguished, as explained by Equation (5). 

### 3.1. Physical Parameter Changes and Outlook

**Figure 4:**
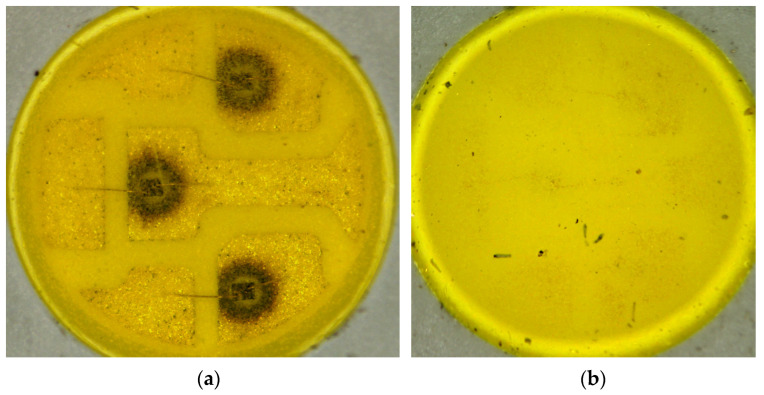
5050-type SMD cool white (**a**,**b**) warm white LED after 150 h of 85/85 ageing (85 °C, 85%RH). The round-shaped dark areas (**a**) are around the chips and follow the silhouettes of the overlapping area of the die attach glue and the silver finish of the lead frame. (**b**) The warm white one looks practically still intact.

**Figure 5:**
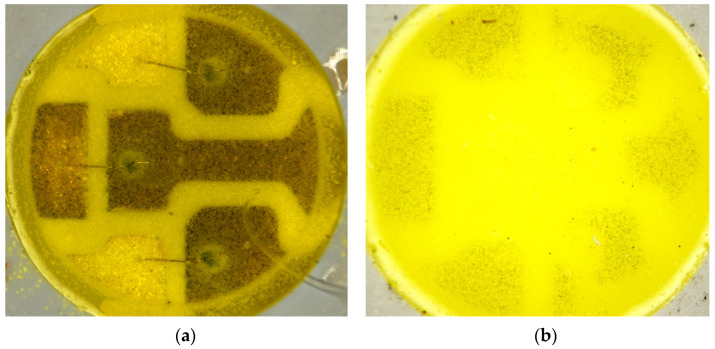
5050-type SMD cool white (**a**,**b**) warm white LED after a 1000 h 85/85 ageing (85 °C, 85%RH). In the cool one, some metallization areas are strongly corroded (**a**), and the surface of the silver finishes may be oxidized. The phosphor/polymer composite is practically transparent because of its degradation. (**b**) On the warm white LED, the degradation of the mould material is much less.

The electrical, thermal, and optical parameters of the investigated LEDs before and after 1000 h of ageing are summarized in Table 1. (Parameter tolerances and accuracy are calculated around max. 5%, not indicated at the values using this manufactured and commercially acquired set of devices). Optical microscopic images of the pcW LEDs in various ageing stages are compared in Figure 4 and Figure 5.

The most important observations regarding pcW LEDs presented in Table 1 can be summarized as follows:

- The electrical “diode” behaviour of the parameters did not change significantly;
- The junction to ambient thermal resistance changed slightly in the range of accuracy in the case of warm white (WW) LEDs, but there was a significantly large increase in the case of cool white (CW) LEDs, which could be explained by the degradation (or delamination) of the chip attachment glue, as described elsewhere [17,37]. The reason could be that in the case of pcCW LED the moisture ingress reaches the chip and under-laying attachment glue level. The junction temperature also increased following similar large differences;
- Similar pronounced differences can be recognized in other parameters: in the case of CW LEDs, the dissipated (thermal) power increased significantly and a strong decrease in the radiant flux (emitted optical power) was detected at the same time. This may be the result of the degradation of the chip [3,5], that of the phosphor, or the darkening of the transparent and reflective layers [2,4,5,21]. The devices have to be regarded as “failed”, luminous flux maintenance shrunk to 62% as opposed to the commonly used 70% level as the failure threshold. These degradations are much smaller or negligible in the case of the WW LEDs, and the luminous flux maintenance remained higher than 90% even after 1000 h of 85/85 ageing.

**Figure 6:**
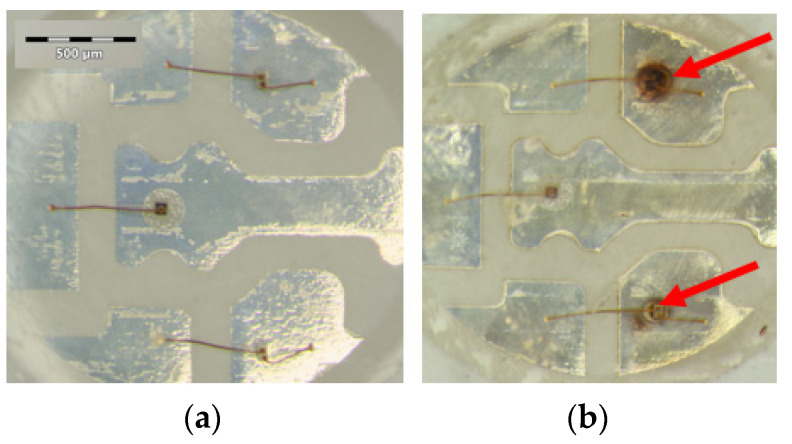
5050-type RGB SMD LEDs after 85/85 ageing (**a**) after 150 h, no changes can be recognized yet, while after 1000 h (**b**) strong brown rings are available around the attachment glue of green and blue LEDs shown by the red arrows; the red’s conducting glue is not corroded even though it may contain silver.

In connection with RGB LEDs, Table 2 and Figure 6 present our findings; the most important observations can be summarized as follows:

- The electrical characteristics practically did not change;
- The thermal resistance from the chip to the environment (ambient temperature) of the red and green chips changed only slightly, while in the case of the blue chip, a significant (60%) increase was measured but the junction temperatures (at room temperature test conditions) increased only by a few centigrade only; the reason could be that in the case of pcCW LED, the moisture ingress reaches the chip and under-laying attachment glue level;
- Despite the thermal resistance changes, the emitted total luminous and radiant fluxes suffered very small changes of a few percent or even zero in the case of the blue chips. The reason is explained generally by the rearrangement of interstitial dopants and/or dislocation movement to the surfaces in the single crystal compound semiconductor materials of the LED chips. The degradation of the chips themselves cannot be observed during the investigated ageing period;
- No changes was recognized after 150 h of 85/85 ageing, while after 1000 h, strong brown rings were formed around the die attach glue of the green and blue LEDs, indicating some kind of degradation (may be carbonization of the epoxy component of the heat conducting insulating composite glue). The red LED’s conducting glue was not corroded even though it may have contained silver.

### 3.2. Changes in LEDs’ Spectra

**Figure 7:**
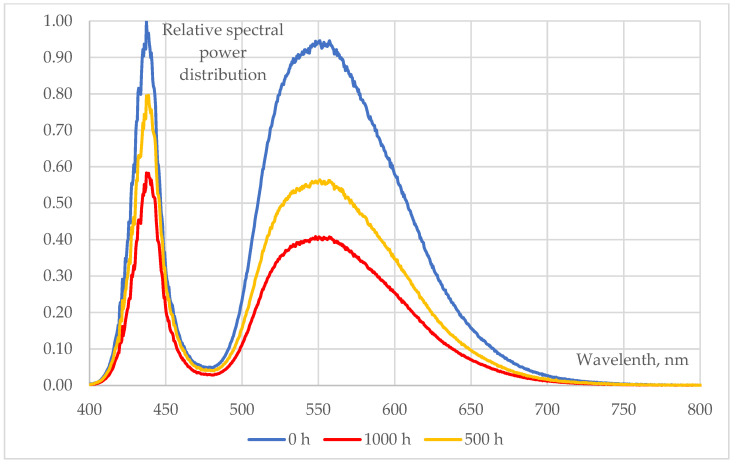
Changes in the relative spectral power distributions of cool white 5050 SMD LEDs (measured at T_a_ = 25 °C, I_total_ = 30 mA) during a 1000 h 85/85 ageing test.

**Figure 8:**
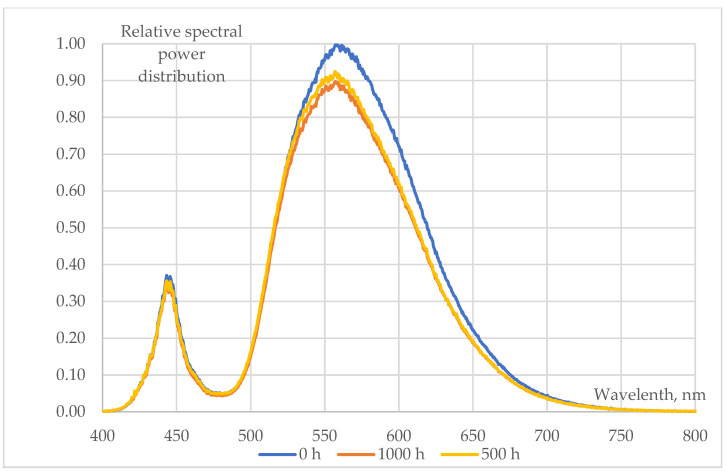
Changes in the relative spectral power distributions of warm white 5050 SMD LEDs (measured at T_a_ = 25 °C, I_total_ = 30 mA) during a 1000 h 85/85 ageing test.

**Figure 9:**
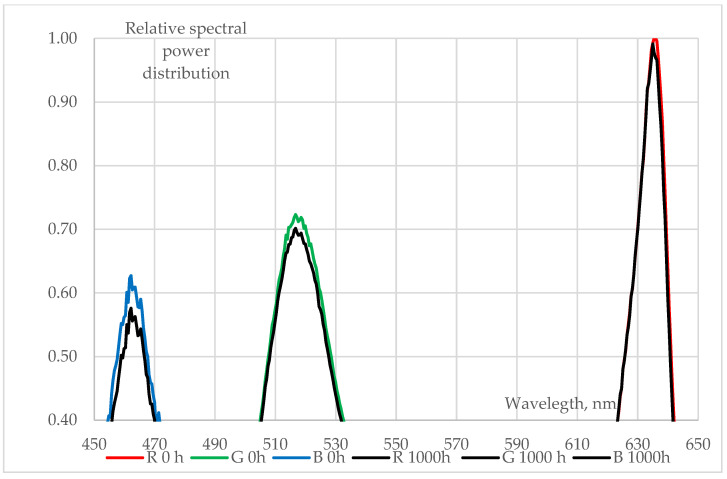
Details of the changes of relative spectra of RGB 5050 SMD LED chips (measured at T_a_ = 25 °C, I_total_ = 30 mA) during a 1000 h 85/85 ageing test (zoomed view at the peaks of the blue, green and red spectra).

Spectral changes of various types (pcCW, pcWW, RGB) of 5050-package type SMD LEDs (measured at T_a_ = 25 °C, I_total_ = 30 mA) during a 1000 h long 85/85 ageing test are shown in Figure 7, Figure 8 and Figure 9:

- The behaviour of phosphor-converted cool white (pcCW) LEDs is shown in Figure 6. The spectral intensities showed a significant decrease in both the blue (blue chips are used for excitation) and the yellow peak, indicating that the degradation may be present not only in the phosphor/polymer mould plastic (maybe mainly in the phosphor grains), but also at chip level at the blue die;
- A more moderate degradation was evidenced at the phosphor-converted warm white (pcCW) LED that is restricted mainly to the yellow phosphor. Since the ratio of the yellow/blue peak ratio is much larger, a larger phosphor grain concentration could be present within the mould material. No degradation of the blue pump chips was experienced in this case;
- Only very small changes in the blue, green and red LED spectra can be revealed, practically in the order of magnitude of the measurement uncertainty of the spectroradiometer that was used. This means that no degradation should be assumed either at the chip level or within the mould material, even though in Figure 5 some degradations can be seen at the insulating die attach glue material.

### 3.3. Luminous Loss

**Figure 10:**
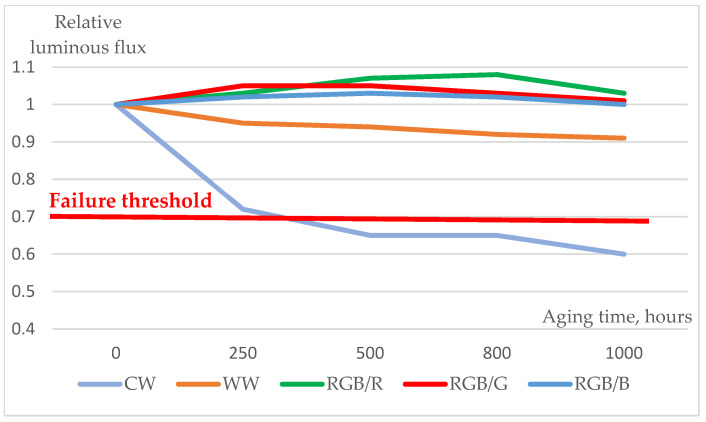
Relative luminous flux maintenance output changes (related to their initial value) of various RGB 5050 SMD LED devices (measured at T_a_ = 25 °C, I_total_ = 10 mA at white and I_total_ = 20 mA at colour chips—as was recommended for their application in an LED string) during the 1000 h 85/85 ageing test.

The ageing time dependence of the relative luminous flux values (aka luminous flux maintenance) of various RGB 5050 SMD LEDs (measured at T_a_ = 25 °C, I_total_ = 10 mA at white and, I_total_ = 20 mA at colour chips—as recommended for their application in an LED string) during 1000 h of the 85/85 ageing test is illustrated in Figure 10. In the case of colour RGB devices, after a slight increase, they maintained their original fluxes. PcWW LEDs showed a definite but limited decrease below 10%. Regarding the large acceleration factor of the applied harsh circumstances, this could be an acceptable level, suggesting that the lifetime in the real applications should probably be far over 10,000 h. Note that no LM80-TM21-compliant lifetime prediction was possible for these devices since we had only a smaller LED population and no actual LM80-compliant ageing was performed. Note also that the pcCW LEDs showed not only significant but very fast degradation (about 40% loss of their initial flux after 1000 h, reaching about 30% loss already after approximately 300 h), which implies a very short lifetime. This suggests that a large ratio of a population of such LEDs may malfunction under real-life conditions much earlier than 10,000 h.

## 4. Discussion: Structural and Material Analysis

**Figure 11:**
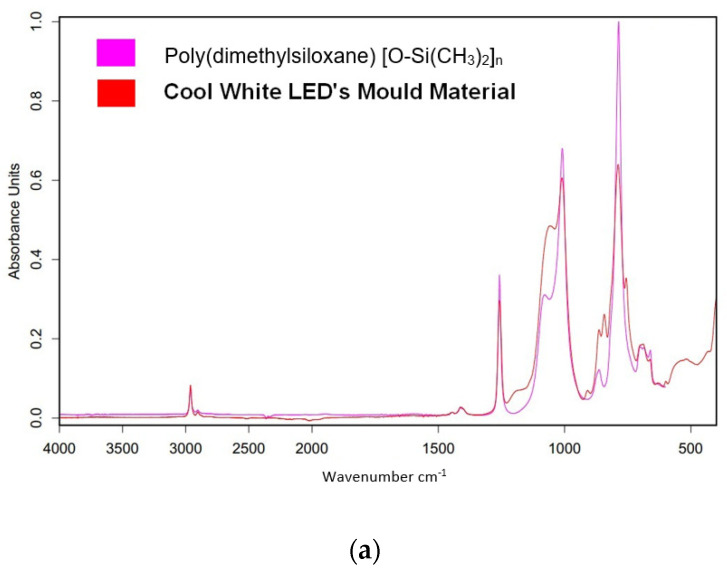

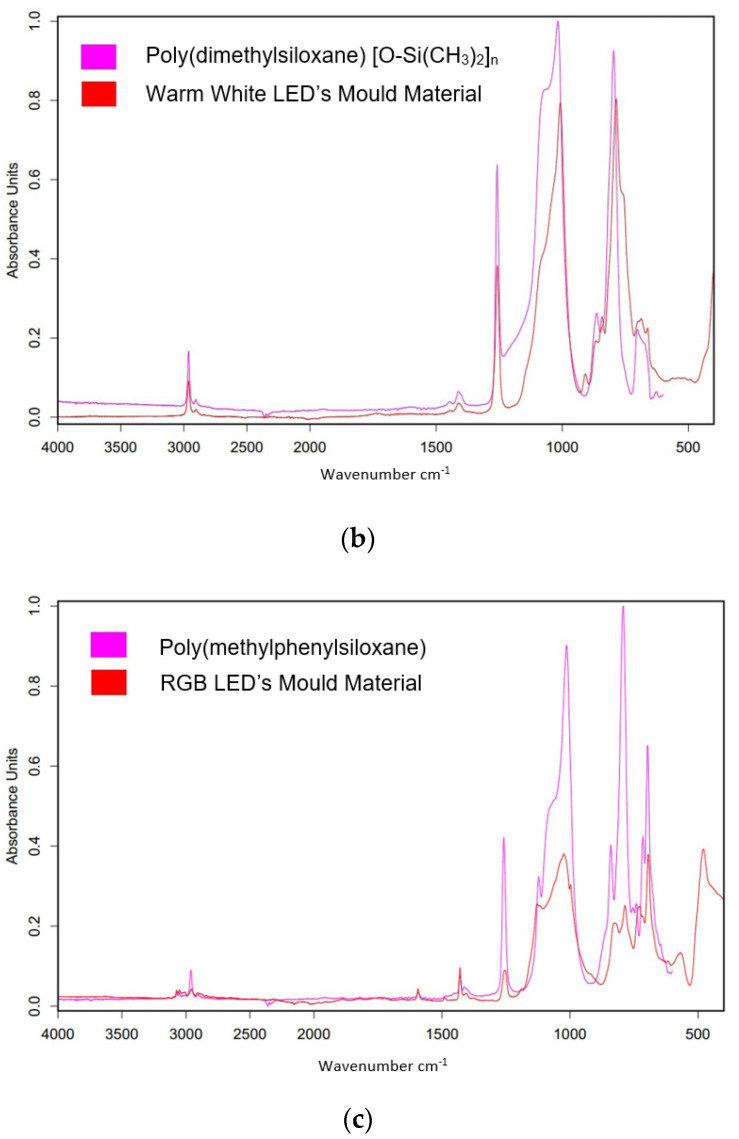
FTIR analysis results on the mould plastic materials of various LED types: (**a**) pcCW and (**b**) pcWW—best matching to poly(dimethyilsiloxane) (PDMS); (**c**) RGB—best matching to poly(methyilphenylsiloxane) (PMPS).

The experimental results shown above suggest that the RGB LEDs are much less sensitive to humidity ingress than the pcW ones. One reason could be that the water molecule absorption and diffusion into the protective mould material would be different. Therefore, an FTIR (Fourier Transform Infrared Spectroscopy) analysis using Brucker Tensor II FTIR equipment was performed on the materials. The results are shown in Figure 11.

The results shown in Figure 11 demonstrate that the RGB and pcW LED types use different polysiloxanes as mould materials; in the case of the RGB LED packages, the transparent poly(methyilphenylsiloxane) (PMPS) was identified, which proved good withstanding against moisture absorption, while in the case of the pcW LED packages, the well-known poly(dimethyilsiloxane) (PDMS) was identified as the phosphor/plastic composite mould, which is much less withstanding against moisture absorption. This explains why the pcW LED packages showed faster degradation in high-humidity ageing environments.

These reliability results complement the information missed by Singh and Tan in their statement that LEDs using phosphor are not as reliable as expected as observed in this work. RGB LEDs could be a better option [21]. 

The other important conclusion from the results of RGB LEDs is that chip level ageing can be neglected in the first 1000 h, and sometimes an increase can even be experienced. These results coincide with previously published works that during the first few hundred or even a few thousand hours of operation; in many cases, the radiated power and luminous flux can still be seen to increase [1,2,3,24], as also cited in the introduction (this phenomenon is explained with the thermally activated rearrangement processes within the semiconductor material: diffusion of vacancies, dopants, and dislocations).

**Figure 12:**
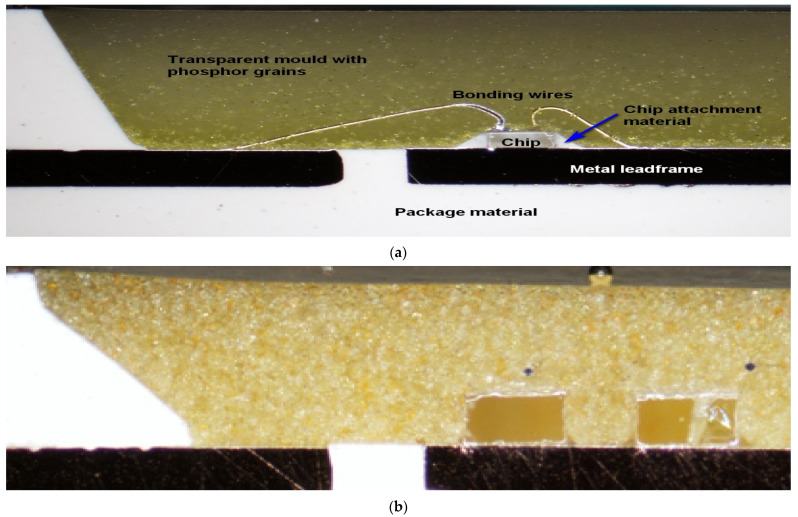
Microscopic image of the cross-sectional views of (**a**) cool and (**b**) warm white LEDs; the difference in the phosphor grain concentration is obvious.

**Figure 13:**
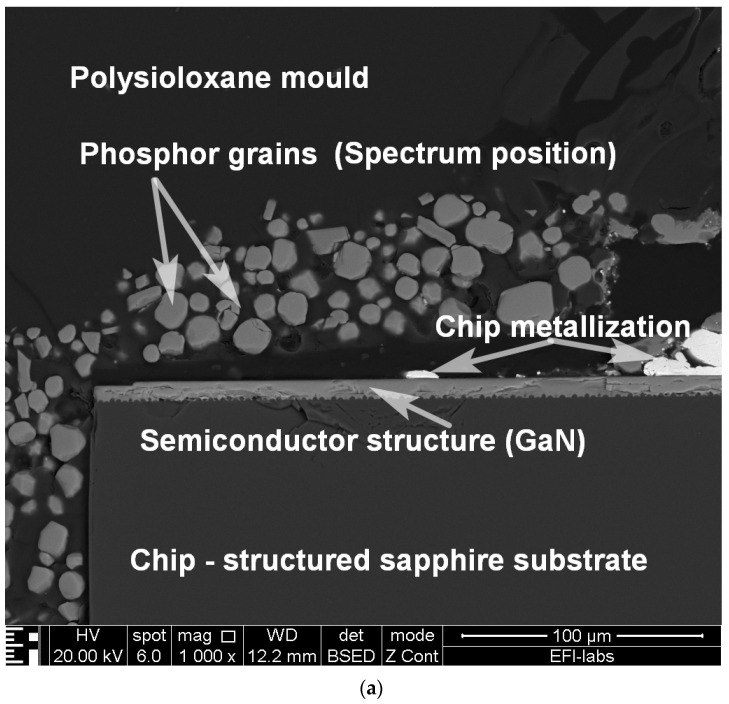

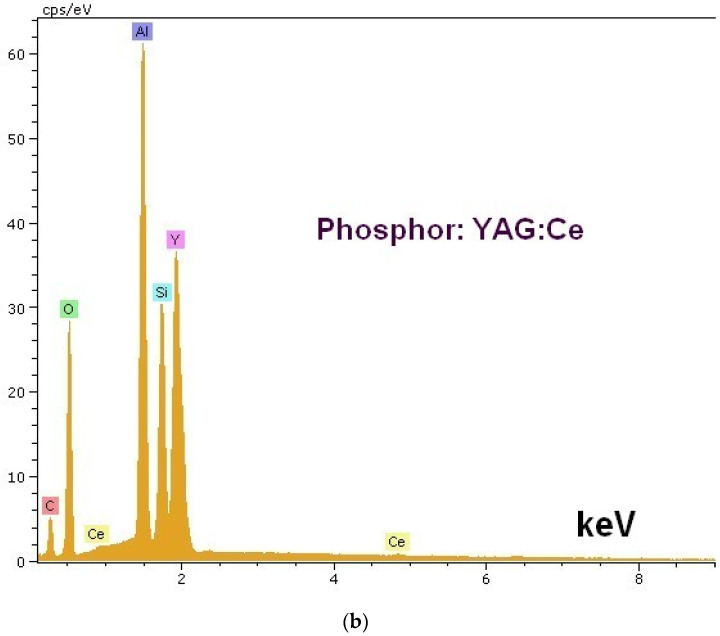
(**a**) Scanning electron microscopy image of the cross-sectional views of the pcCW LED; the parts are indicated in the picture. (**b**) X-ray spectrum of the phosphor grain.

The third obvious characteristic difference was between the degradation speeds of the two white types. Therefore, an analysis was performed on their structure, which is illustrated in Figure 12. The figure demonstrates that both the concentration and the distribution of the phosphor grains are different: it is relatively large and homogenous in the CW LEDs’ mould layer, while a low amount of phosphor is deposited on the bottom of the pool in CW LED, practically on the chip surface and surrounding areas. It seems that the larger concentration of phosphor not only provides a relatively lower concentration of degraded phosphor amount, degraded by practically the same amount of diffusing water molecules, but also may protect the lower levels from degradation. This, again, also strengthens the experience of Shingh and Tan [21], suggesting that the presence of phosphor in white LEDs may provide some kind of protection for the LED chip and the underlying layers of phosphor/polysiloxane composite from moisture. To study this possibility further, an analysis of the phosphor grains was also performed. The results are shown in Figure 13.

The results of the scanning electron microscopy (SEM) and related eectron probe X-ray microanalysis are shown in Figure 13. The parts of the structure are designated on the SEM image: these are both results of spectroscopy analysis but also obvious from previous studies [1,2,3,4,5,6,7,8,9,10]. The spectrum of the phosphor grain shows that their row material is YAG:Ce, which is used in many white LED applications. The presence of C, Si, and O elements in the spectrum is scattered from the polysiloxane mould matrix material. 

It is well known that Ce could react with moisture according to the following reaction scheme [38]:

2 Ce + 6 H_2_O → 2 Ce(OH)_3_ + 3 H_2_ (6)

Thus, the phosphor can consume the diffusing water molecules and protect underlying films and chips when it is in a layer that is thick enough.

**Figure 14:**
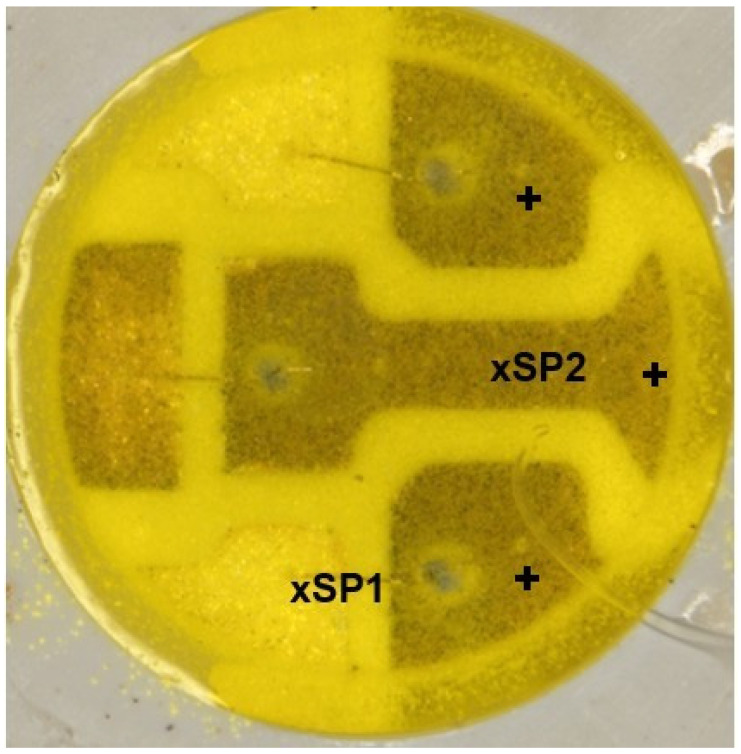
Microscopic image of the 5050-type SMD cool white LED with strongly corroded metallization surfaces. After de-capsulation, electron probe X-ray microanalysis was performed on the designated spots. (+ designates the anode).

As can be seen in Figure 5a, the moisture reached the bottom of the cool white LED’s mould pool and resulted in corrosion of the bottom metallization. De-capsulation and electron probe X-ray microanalysis were performed on the LED, and the results are shown in Figure 14 and Table 3.

It can be seen that the silver/oxygen ratio is much larger in the intact areas, so the formation of silver oxide can be supposed in the corroded areas. (The other elements are present in the multilayer lead frame metallization with silver finishes (Fe, Ni), Ti, is a filler material in the packaging plastic, while C, Si and also O are present in the polysiloxane mould material residues). The formation of silver oxide in the presence of moisture follows the next well-known reaction scheme if the metal surface is on bias potential [38]:

Ag → Ag^+^ + e^−^, Ag^+^ + OH^−^ → AgOH, 2AgOH → 2Ag_2_O + H_2_O (7)

The strongly corroded areas are indeed on the anodic sites. (One of the cathodes was also somewhat corroded, the reason for which may be in the different polarization conditions).

## 5. Conclusions

The climatic reliability of LEDs was studied concentrating mainly on the effect of high humidity. Thermal humidity bias (THB—85 °C/85%RH) ageing was performed on various LED types. The experimental results presented here showed that the differences stem from their material composition and structure. The following main conclusions can be drawn:

- RGB LEDs are much less sensitive to humidity ingress than the phosphor-converted white (pcW) LEDa. The reason is that the water molecule absorption and diffusion into the protective mould material are different; the PMPS protective material in RGB LEDs is much more withstanding against moisture absorption than the PDMS/phosphor composite in pcW types;
- The behaviour of phosphor-converted cool white (pcCW) and warm white (pcWW) was also different. The WW LED showed much less degradation during the ageing tests we performed, though the resulting luminous flux depreciation still might be acceptable subject to real-life mission profiles during their life span in LED-based illumination systems. CW LEDs have shown very fast and dramatic degradation leading to early parametric failure. The difference in their material structure is the concentration of the phosphor material within the plastic/phosphor composite layer. In WW LEDs, this layer has a large concentration and homogeneous distribution of the phosphor grains, while in CW LEDs only the bottom layer just above the chips contains phosphor;
- The applied phosphor material in pcW LEDs (YAG:Ce) can react with water molecules and protect the bottom levels from moisture ingress in the WW LEDs. At the same time, the water molecules can reach the bottom level of the plastic/phosphor pool in the CW LEDs and thus result in chip-level degradation, lead frame metallization corrosion, and the degradation/delamination of the heat-conducting glue under the chips;
- As a final conclusion, it could be stated that in this experimental work, colour (RGB) LEDs were the most reliable and phosphor-converted cool white (pcCW) LEDs were found to be the most unstable in high-humidity environments.

Hopefully, these results may contribute to LED material development processes. Further research is needed to study their behaviour in other ageing processes and other LED types should also be investigated since the applied materials may vary in other types.

## Figures and Tables

**Table 1:** The electrical, thermal, and optical parameter changes of the selected 5050-package-type phosphor-converted white LEDs during an 85/85 test (measured at T_amb_ = 25 °C, forward current 30 mA for the package, 10 mA pro chip, tolerance of the measurements and sample deviations within 5%).

	Cool White		Warm White	
Parameter	Original Value	After 1000 h of 85/85 Ageing	Original Value	After 1000 h of 85/85 Ageing
Forward voltage [V]	3.1	3.1	2.9	2.9
Electrical power [mW]	92	92	87	87
Heat dissipation [mW]	77	82	62	64
Emitted optical power [mW]	15	10	25	23
Energy conversion efficiency [%]	16	11	29	26
Thermal resistance [K/W]	211	455 (∆ = −115%)	115	117 (∆ = −1.7%)
Junction temperature [°C]	42	62	32	32
Total luminous flux [lm]	4	2.5 (∆ = −38%)	9.1	8.3 (∆ = −9%)
Efficacy lm/W	43	27	105	95.4
Correlated Color Temperature [K]	11,900	4340	5315	5676

**Table 2:** The electrical, thermal, and optical parameter changes of the selected 5050-package-type RGB LEDs’ different coloured chips during the 85/85 test (measured at T_a_ = 25 °C, forward current 20 mA pro chip tolerance within 5%).

	Red		Green		Blue	
Parameter	Original Value	After 1000 h of 85/85 Ageing	Original Value	After 1000 h of 85/85 Ageing	Original Value	After 1000 h of 85/85 Ageing
Forward voltage [V]	2.1	2.1	3.3	3.3	3.2	3.2
Electrical power [mW]	42	42	66	66	64	64
Heat dissipation [mW]	36	36	59	59	54	54
Emitted optical power [mW]	6	6	7	7	10	10
Energy conversion efficiency [%]	14	14	11	11	18	18
Thermal resistance [K/W]	254	280 (∆ = −10%)	277	307 (∆ = −11%)	235	375 (∆ = 60%)
Junction temperature [°C]	34	35	41	43	37	41
Total luminous flux [lm]	1.42	1.46 (∆ = −3%)	3.01	3.05 (∆% = 1%)	0.55	0.55 (∆ = 0%)
Efficacy [lm/W]	34	35	45	46	8.5	8.5

**Table 3:** Quantitative electron probe X-ray microanalysis results at the locations designated in Figure 14; these analyses were performed on the designated spots.

Atomic Percent (%)	C	O	Si	Ti	Fe	Ni	Cu	Ag
LED SP1	8.96	17.40	1.76	2.34	2.66	10.68	9.36	46.85
LED SP2	9.07	33.63	1.97	2.78	1.73	8.17	7.30	35.34

## Data Availability

The raw/processed data required to reproduce these findings cannot be shared at this time as the data also form part of an ongoing study.
